# Prediction of LC-MS/MS Properties of Peptides from Sequence by Deep Learning*[Fn FN2]

**DOI:** 10.1074/mcp.TIR119.001412

**Published:** 2019-06-27

**Authors:** Shenheng Guan, Michael F. Moran, Bin Ma

**Affiliations:** ‡ David R. Cheriton School of Computer Science, University of Waterloo, Waterloo, ON, N2L 3G1, Canada; § Program in Cell Biology and SPARC BioCentre, Hospital for Sick Children, 686 Bay St, Toronto, ON, M5G 0A4, Canada; ¶ Department of Molecular Genetics, University of Toronto, 686 Bay St, Toronto, ON, M5G 0A4, Canada

**Keywords:** Algorithms, Peptides, Statistics, Bioinformatics Software, Bioinformatics, Charge State Distribution, Deep Learning, Indexed Retention Time, Prediction

## Abstract

Deep learning models for prediction of three key LC-MS/MS properties from peptide sequences were developed. The LC-MS/MS properties or behaviors are indexed retention times (iRT), MS1 or survey scan charge state distributions, and sequence ion intensities of HCD spectra. A common core deep supervised learning architecture, bidirectional long-short term memory (LSTM) recurrent neural networks was used to construct the three prediction models. Two featurization schemes were proposed and demonstrated to allow for efficient encoding of modifications. The iRT and charge state distribution models were trained with on order of 10^5^ data points each. An HCD sequence ion prediction model was trained with 2 × 10^6^ experimental spectra. The iRT prediction model and HCD sequence ion prediction model provide improved accuracies over the start-of-the-art models available in literature. The MS1 charge state distribution prediction model offers excellent performance. The prediction models can be used to enhance peptide identification and quantification in data-dependent acquisition and data-independent acquisition (DIA) experiments as well as to assist MRM (multiple reaction monitoring) and PRM (parallel reaction monitoring) experiment design.

## Abstract (precis)

Indexed retention times (iRT), MS1 (the first level of mass analysis) or survey scan charge state distributions, and sequence ion intensities of MSMS (tandom mass spectrometry) spectra were predicted from peptide sequence by use of long-short term memory (LSTM) recurrent neural networks models. Data points on the order of 10^5^ were used to train the iRT and charge state distribution models. An HCD sequence ion prediction model was trained with 2×10^6^ experimental spectra. The models with a simple deep learning architecture can predict those three key LC-MS/MS (Liquid chromatography-tandem mass spectrometry) properties with superior accuracies.

## Graphical Abstract

**Figure:**
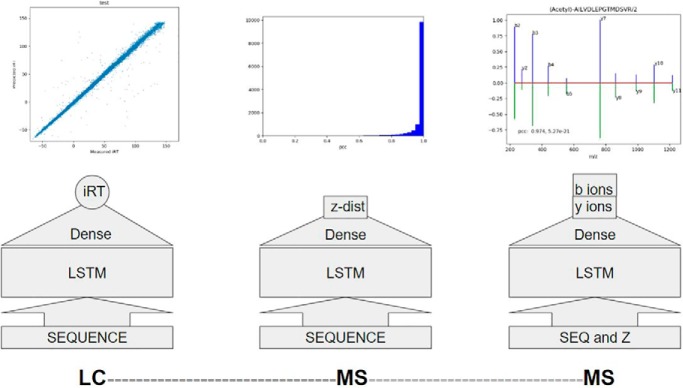

**Highlights**

- Deep learning models for prediction of LC-MS/MS properties.

The ability to directly predict LC-MS/MS^1^ behaviors from peptide amino acid residue sequence will power the next generation of proteomics research. Currently, the major behavior or properties of peptides in the context of LC-MS/MS are obtained experimentally, suffering from high cost and inhomogeneous accuracies. This work intends to address those issues directly using a common deep learning model architecture trained with experimental data to predict useful LC-MS/MS properties, such as indexed retention times (iRT), MS1 or survey or precursor scan charge state distributions, and MS/MS sequence ion intensities. Those properties can be directly used for enhancing peptide identification and quantification in both data-dependent acquisition and data-independent acquisition (DIA) experiments as well as for designing MRM and PRM experiments.

The previous research into prediction of those three major classes of LC-MS/MS properties varies significantly. The first use of machine learning algorithms for retention time prediction was with simple dense neural networks (1). Since then, many algorithms have been proposed (2). DeepRT is the most recent method utilizing a deep learning model to predict retention time (3). It is surprising that information of charge state distributions of MS1 scans has not been extensively used to facilitate peptide identification and quantification. Studies for prediction of charge state distributions were carried out with simple models and limited data (4, 5). Prediction of MS/MS spectra is both theoretically interesting and practically useful. Development of the kinetic models by Zhang (6–9) requires deep understanding of ion fragmentation mechanisms. The kinetic model development is a reminiscence of knowledge-based artificial intelligence in proteomics. Like in the other fields, such as image processing and language processing, deep-learning-based approaches in the field of proteomics quickly outperform any knowledge-based approaches. DeepNovo (10), a deep learning model based *de novo* sequencing method provides an accurate peptide sequencing strategy without the reference to a predetermined protein sequence database and digestion rules. The deep-learning-based *de novo* sequencing method has been extended for DIA experiments (11). The recently developed pDeep algorithm allows for prediction of MS/MS spectra with peptide sequences without incorporating detailed fragmentation mechanisms into the model (12). Schoenholz *et al.* (13) developed a similar long-short term memory (LSTM)-model-based search algorithm called “DeepMatch.” Gessulat *et al.* (14) recently developed a deep-learning-based tool called “Prosit” for prediction of LC retention time and fragment ion intensities from sequences of synthetic peptides. Tiwary *et al.* (15) reported two deep learning models for MS/MS spectrum prediction.

The core motivation of this work is to identify the key LC-MS/MS properties that are determined solely by peptide sequence. The chosen three properties fit the criteria. The iRT calibration strategy (16) allows establishment of a common scale to calibrate experimental retention times. Solvent gradient, column temperature, sample loading, and other experimental conditions can be incorporated into the iRT *versus* RT (retention time) or RT *versus* iRT calibration functions. MS1 charge state distributions may change with many experimental conditions. One of the most important ones is the charge detection method of the mass analyzer (see “Discussion” section). However, given commonly used mass analyzers, MS1 charge state distributions are stable against many other experimental conditions. For MS/MS sequence ion intensity prediction, different ion series may present in an MS/MS spectrum with different fragmentation methods and fragment ion intensities also change with different mass analyzer/detector. Spectral behavior changes also with fragmentation conditions such as activation energy and activation duration. However, MS/MS spectral behavior is remarkably stable under commonly used instrument classes and standard methods. Under the constraints discussed above, we demonstrate in this work that practical deep learning models can be trained to predict those key LC-MS/MS properties with excellent accuracies.

Since the beginning of this decade, neural-network-based deep learning models have made significant impact on image and natural language processing (17). For peptide property prediction, recurrent neural network models, such as LSTM models, are a natural choice because they are able to handle sequential input data with a variable length, such as peptide sequences (in spectrum prediction, charge state is also an input). Deep supervised learning models are capable of classification and regression tasks. Our iRT and spectrum prediction models are classic regression examples, but the charge state prediction model can also be considered as a classification method in which the output is charge state classification probabilities. The three prediction models demonstrated here represent three levels of data in LC-MS/MS: iRT for LC behavior, charge state distribution for MS1, and HCD sequence ion intensities at MSMS level. They also differ in output dimensionality: iRT is a scalar, charge state distribution is a vector, and HCD sequence ion intensities are two-dimensional matrix with one of the dimensions dependent on the peptide length. However, the core learning blocks are the same bidirectional LSTM layers, demonstrating the applicability of deep learning models for a wide range of problems. As in other fields where deep learning made significant impacts, performance of peptide property prediction models critically depends on the availability of large quantity and high-quality data. For example, our HCD sequence ion intensity prediction model was built on a large pool of spectra of excellent quality (18), collected from the whole proteomics community.

## EXPERIMENTAL PROCEDURES

### 

#### 

##### Sources of Data

iRT data: Data used for training the iRT model were described in (19). The data were compiled from three DIA technical runs with HeLa and HEK293 cell lysates, with 1-m long column and 4-h acquisition time. According to Bruderer *et al.* (19), the identification false discovery rate was controlled at 1%. Peptides of duplicate identifications and different charge states were pooled together, and the pooled median iRT values were used. After filtering, such as limiting the peptide length to 40 or less, the total number of peptides for the iRT model is 125,793, in which 10% were randomly selected as the test dataset, and the rest 90% were used to train the iRT model.

Charge state distribution data: For the charge state distribution model, raw data files of data-dependent acquisition runs (19) were downloaded from ProteomExchange. Those data-dependent acquisition experiments included 0.5–4-h single-shot runs and high pH fractionation sample runs. The raw data files were converted to peaklists using previously developed PAVA code (20). MS2 or MS/MS peaklists were searched with the MSGF+ search engine (21). The human proteome (Uniprot Proteome ID: UP000005640) sequences (71,778 entries) were downloaded on February 6, 2018. Trypsin specificity with up to five missed cleavages were allowed (filtered after search). Cysteine carbamidomethylation was the fixed modification. Variable modifications were oxidation of methionine, pyro-glu from peptide n-terminal glutamine, and protein n-terminal acetylation with or without loss of methionine and deamidation of glutamine and asparagine. Precursor mass tolerance was 10 ppm, and fragment ion detection instrument was set to “Q Exactive.” The peptide EValue cutoff of 0.045 was used to obtain the peptide false discovery rate of 1.0%.

For an identified peptide, the ion chromatograms for charge states 1–5 were extracted from survey scans and the XICs (extracted ion chromatograms) were fitted with a polynomial variance Gaussian function (20). The intensities of peptide charge states were normalized by division of their sum. The total number of peptides for the charge state distribution model is 126,876, in which 10% of randomly selected data were used as the test dataset.

HCD sequence ion intensity prediction data: A human HCD spectral library with 2,154,269 peptide ions was compiled by Wang *et al.* (18). Fragment ions were annotated with in-house code, and data were formatted as the National Institute of Standards and Technology (NIST) MSP format. Monoisotopic and isotopic ions for singly and doubly charged b- and y- ions, internal ions, H_2_O and NH3 neutral loss ions, and the precursor and their neutral loss ions were annotated. For precursor ions with charge state of 2, b and y ions of charge state one were collected. For precursors with charge state 3 to 6, both charge states of 1 and 2 were considered for b and y ions. The ion intensities of a single spectrum were normalized by division of the highest intensity. Due to the graphic processing unit, GPU's memory limitation, the dataset was separated into two subsets of 1,074,898 spectra each. The membership of the subsets was random. The HCD prediction model was trained sequentially with the two subsets. Out of the 1,074,898 spectra in the second subset, 10% were used as the test dataset.

For the selected test cases, raw files from Bruderer *et al.* (19) and from ProteomeTools (22) were used.

##### Feature Structure

Peptide sequence features: A peptide sequence is represented with one-hot encoding array of size, MAX_LENGTH X NO_OF_AAS, in which MAX_LENGTH of 40 is the maximum length of peptides and NO_OF_AAS is the number of amino acid types. In all cases, a deamidated glutamine residue is considered as a glutamic acid and a deamidated asparagine as an aspartic acid. In the iRT model, only modification allowed is the oxidized methionine, encoded separately Therefore, NO_OF_AAS is 21 for the iRT model. For the charge state distribution model, oxidized methionine, pyro-glutamine, and n-terminal acetylation were separated feature types. Its NO_OF_AAS is 23.

For the sequence ion intensity prediction model, peptide n-terminal carbamylation was also considered as a separate type, given its NO_OF_AAS of 24. The charge state of a peptide ion (precursor ion) is one-hot encoded as a six-member long array. Therefore, a peptide ion is encoded as a 40 × 30 matrix (20 natural amino acid types, 4 PTM types, 6 charge state types). As suggested by (12), normalized collision energy was not used as a feature. For peptides of length less than 40, zeros were added to both sides (zero padding on both ends). The one-hot encoding of peptide sequence and modification featurization is illustrated in Supplemental Fig. S2.1.

##### Learning Models

All the models consist of a masking layer followed by two stacks of bidirectional LSTM layers. For regularization, both dropout and recurrent dropout were applied. The models differ in output (label) layers: for the iRT model the output was a scalar and no final activation was used. Two dense layers with activation function of hyperbolic tangent function (tanh) were used and the final output has a dimension of one. For the charge state distribution model, there were two dense layers with the output as one dimensional array of size 5 (corresponding to charge states 1 to 5). The output activation function was softmax. For the spectral prediction model, a time distributed dense layer with sigmoid activation function was used. The output (label) structure is illustrated in Supplemental Fig. S2.2.

All LSTM models, implemented with Keras framework (23) were trained on a GeForce RTX 2080 Ti Graphics Card with 11 GB video memory. The longest training time was 7.2 h for one subset (1,074,898 spectra) of the HCD sequence ion prediction model.

## RESULTS

### 

#### 

##### iRT Prediction

**Fig. 1.**
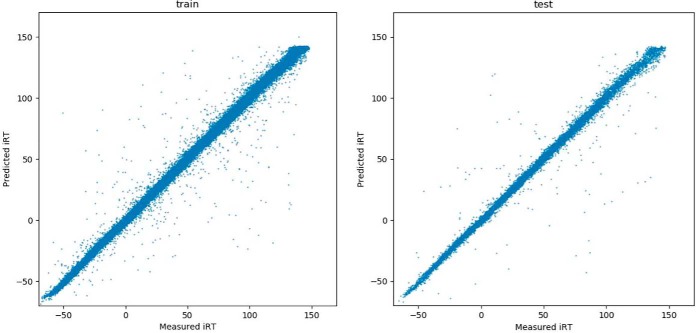
**Scatter plots of measured iRT against predicted iRT.** *Left*: the training dataset. *Right*: the test dataset.

The essence of a deep learning model is to capture the data generation distribution. However, we do not have the independent information about the distribution underlying the iRT dataset. The iRT values were obtained from the DIA experiments (19), and several sources of errors may be incorporated into the dataset. First, the peptide false discovery rate was about 1%, and therefore, at least 1% of the iRT values were incorrect, and those incorrect iRT entries are likely to contribute to outliers. Second, calibration of RT *versus* iRT can introduce some uncertainties (RT *versus* iRT calibration is briefly described in Supplemental Material Section S1). Third, when the iRT values were aggregated from different LC runs, errors are introduced due to LC alignment inconsistencies. As can be observed in the Fig. 1, the outliers are mostly likely due to the incorrect peptide identification. The variation becomes higher with increased iRT values. For peptides of later elution or having larger iRT values, intensities may spread out in more charge states and in wider LC peak shapes, and retention time measurement becomes less accurate.

**Fig. 2.**
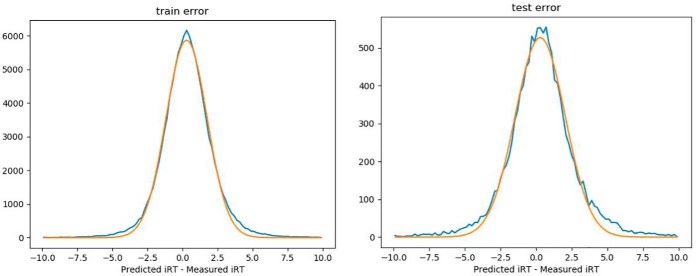
**Distribution of errors for iRT prediction model.** Blue curves are error distributions and orange curves are the corresponding Gaussian fits. *Left*: training errors. *Right*: testing error.

Both training and testing error distributions are close to a Gaussian (Fig. 2), but away from the center there are still significant deviation of the error distributions from the Gaussian-fitted curves. The 95% confidence intervals were computed from distributions numerically, and they are 7.18 and 9.62 iRT units, for training and test datasets, respectively.

A higher capacity model was also investigated with nearly twice as many parameters, and it did not result in any improvement in the confidence intervals, suggesting the current model may have captured the most important characteristics of the iRT data generation distribution. Several different deep learning models have also been surveyed, including conventional CNN (convolutional neural networks), temporal CNN (24), and Capsnet (3). None of the models surveyed performed better than the current LSTM model with the current datasets. It is nearly impossible to obtain an optimal deep model because model hyperparameter optimization is multidimensional and problem dependent. We can, however, make a rough comparison of performance between our model to that of DeepRT(3). Our test confidence interval of 9.62 iRT units (iRT span: −60 to 140) is about 28% narrower than the DeepRT result of 13.4 (iRT span: −40 to 160, Supplemental Fig. S7 of reference (3)) for reverse-phase LC experiments.

##### Prediction of Charge State Distribution

Survey scan or MS1 charge state distribution provides rich information on relative charge partition among peptide ions. The information may also be helpful in assessing the quality of the associated MSMS spectra and may be used directly for extraction of MS1 ion chromatograms. In two previous studies (4, 5), conventional machine learning models were trained to predict charge states for electron transfer dissociation spectra. A common problem with the conventional machine learning algorithms is the requirement for manual collection of features. In both case, features are extracted from identified ETD (electron transfer dissociation) tandem mass spectra. Liu *et al.* (25) trained a model with linear combination of amino acid composition with Gaussian probability. In our model, only peptide sequences were used, and the deep learning model is capable of learning the complex contribution of peptide sequence to the charge state distribution. The relative intensities of different charge states detected in a mass spectrum are not only directly proportional to their abundances. In a Fourier transform ion cyclotron resonance mass spectrometer, the detected image current is inversely proportional to the ion's *m/z* (26). In an Orbitrap, the image current is inversely proportional to the square root of the ion's *m/z* (27). However, for our purpose, treating the relative intensities as one of LC-MS/MS properties is advantageous: the predicted distribution can facilitate peptide identification and quantification. Therefore, although we refer to the term “charge state distribution,” it is an LC-MS/MS behavior other than the true charge partitions on a peptide. The deep model on the other hand is able to learn the “behavior” or property with great accuracy. However, the property should not be transferred to other mass analyzers without proper intensity calibration.

**Fig. 3.**
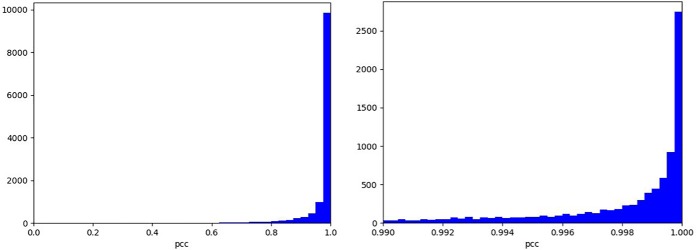
**PCC distribution for charge state distribution prediction *versus* experimental measurement of the test dataset.** The MS1 charge state distribution prediction model was trained with 90% of 126,876 experimental distributions. 10% of those were used as the test dataset to evaluate the model shown in this figure. *Left*: full range. *Right*: zoomed range of [ 0.99, 1].

As shown in Fig. 3, the charge state distribution prediction produces highly accurate results judged by the test Pearson correlation coefficient (PCC) distribution, with median PCCs of 0.998 and 0.997 for the training and testing datasets, respectively. The distribution for the test dataset is similar to that for the training dataset, indicating that overfitting is not a significant issue here.

**Fig. 4.**
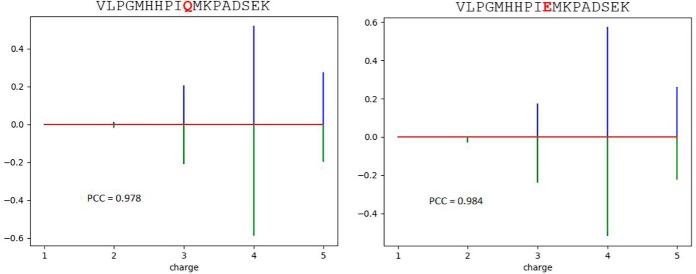
Charge state prediction of peptide VLPGMHHPIQMKPADSEK (*right*) and its Q-deamidated form (*left*). The upper-pointing sticks (blue) are measured data, and the lower-pointing sticks (green) are the predicted values.

Two examples of charge state prediction are illustrated in Fig. 4. For peptide VLPGMHHPIQMKPADSEK and its Q-deamidated form, the charge state distributions are quite similar. The missing observed charge state of 2 for the deamidated form may be due to its low abundance for experimental detection.

##### Prediction of HCD Sequence Ion Intensities

The whole dataset for training the spectrum prediction model contains two subsets of 1,074,898 HCD spectra each. The HCD prediction model was trained sequentially with the two subsets. 10% of spectra in the second subset were used as the testing set. During training, 20% of the training subsets were used to validate the model. In the whole dataset, 3.85% contains n-terminal acetylation, 8.10% methionine oxidation, 2.00% pyro-glutamine, and 5.68% n-terminal carbamylation.

**Fig. 5.**
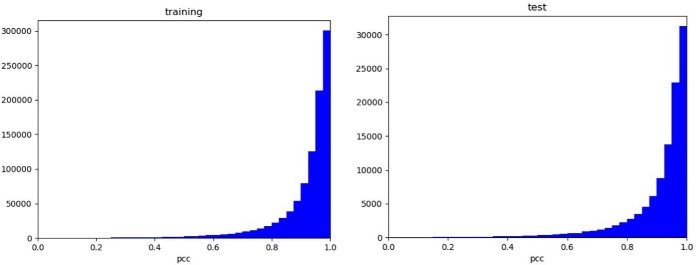
**Distributions of PCC between experimental and predicted HCD sequence ion intensities.** *Left*: for training dataset; *Right*: for testing dataset.

As shown in Fig. 5, the PCC distributions for training and testing are also quite similar, indicating that the model does not overfit much. The median PCCs are 0.955 and 0.953 for training and testing, respectively. The median test PPC for our test dataset is slightly better than that obtained in the pDeep study (12). A breakdown analysis of performance is provided in Supplemental Table S3. For peptide ions of 20 amino acid residues or shorter with no modifications, the median test PCC of 0.965 is a more significant improvement from that (0.950) for the pDeep model. However, our dataset contains peptide sequence length up to 40 amino acid residues compared with 20 in the pDeep study. For human proteome, this adds about 25% more unique peptides (see peptide length distribution of human protein sequences in Supplemental Fig. S3). In addition, the pDeep data do not contain modified peptides, and in our dataset, peptides with the following modifications are allowed: oxidized methionine, peptide n-terminal pyro-glutamic acid, peptide n-terminal carbamylation, and protein n-terminal acetylation. In addition to increase sequence length and inclusion of some common modifications, our model differs from that of pDeep in the number of features: our model uses fewer features. One of the core advantages of deep learning over traditional machine learning is that it uses multiple layers to learn feature characteristics. Therefore, deep models are typically much simpler in terms of feature engineering (feature collection, normalization, reduction, etc.). We designed our models using the simplest possible architecture with only 30 feature dimensions (to represent 20 natural amino acid residues, 2 modified amino acid residues, 2 separately coded modifications, and 6 charge states) *versus* 88 in the pDeep model (illustrated in Supplemental Fig. S2.1). Our model also aligns n-terminal (b) and c-terminal (y) ions with the peptide sequence (see Supplemental Fig. S2.2 for an example of model label (output) structure), whereas in the pDeep model the length (19) of the series of ions is one less than that (20) of the peptide sequence length.

One of the utilities of HCD sequence ion intensity prediction is to assist isomeric peptide assignments. Isomeric peptides concerned here are peptides with exactly the same chemical composition and nearly identical sequences. The minor difference in sequence makes them difficult to be distinguished using MS2 spectra. The authors of the pDeep model (12) investigated their model's utility to distinguish three classes of the extremely similar peptides: (a) substitution of an isoleucine for a leucine; (b) shared chemical formula of different amino acid combinations, such as GG for N; and (c) local amino acid permutations, such as AF for FA.

**Fig. 6.**
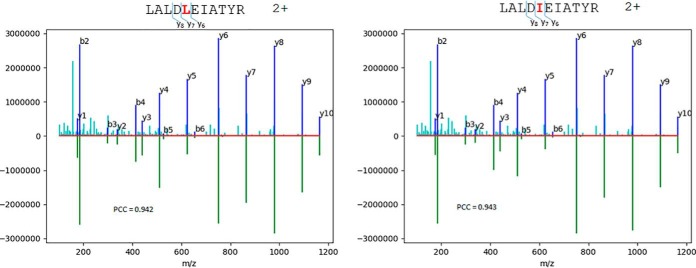
Comparison of observed HCD spectrum and predicted sequence ions from two isomeric sequences differed by leucine (*left*) or isoleucine (*right*) at position 4. The low portions are predicted HCD sequence ions and the upper portions are the same experimental spectrum, Peaks marked with a dark blue color are annotated, while unannotated peaks are in light blue. A noticeable change in relative intensity of y_6_ ions can be observed. However, the difference is much more quantifiable with a “local” ion PCC.

In Fig. 6, an experimental HCD spectrum was identified by the MSGF+ search engine as LALDLEIATYR 2+ ion of the keratin, type II cytoskeletal 8 (P05787) or LALDIEIATYR 2+ ion of the keratin, type II cytoskeletal 1 (P04264). Because both sequences were assigned with the same EValue (4.2 × 10^−6^, 1% false discovery rate cutoff = 0.045), the search engine was not capable of distinguishing them. When the experimental spectrum was matched with the predicted ion intensities of the two sequences, the PCC difference for matching between the experimental and predicted data was small (0.942 for LALDLEIATYR and 0.943 for LALDIEIATYR) if all sequence ions were used. However, if only some “local” ions were considered, such as y_6_, y_7_, and y_8_, there is a clear difference in the relative intensities. The “local” ion PCC values for the three ions were 0.870 and 0.994 for LALDLEIATYR and LALDIEIATYR, respectively. Clearly, using the local product ions near the site of sequence difference can provide more significant discrimination power. The assignment of the isoleucine peptide of LALDIEIATYR was also supported by spectral count information. In the same raw data file, there are 137 spectra including those for the isoleucine peptide (four spectra) belonging to the protein (P04264), whereas only 11 spectra supporting the protein (P05787) contain the leucine peptide. Four out of the 11 spectra were assigned to the leucine peptide. It is unlikely that the experimental spectrum is a mixture of both peptides because they also have different retention times. Both the matching analysis of predicted ion intensities and the spectral counting information support the assignment of the isoleucine peptide of LALDIEIATYR. Another example for leucine/isoleucine peptide discrimination is provided in Supplemental Fig. S4.1. The “local” ions with the most discriminating power can be selected (i) by choosing ions near the site of difference and (ii) by examining local PCC values between the predicted ion intensities of the isomeric sequences.

The isomeric peptide classes (b) and (c) can be easily distinguished if a fragment ion is observed for the peptide bond cleavage between amino acid residue combination or permutation. If this is not the case, the predicted ion intensity difference may be used to distinguish the assignment. For class (b), the pDeep model was able to provide strong support for assignment of an experimental spectrum from the ProteomeTools dataset to the peptide GGFFSFGDLTK (PCC = 0.98) against NFFSFGDLTK (PCC = 0.87). Using our HCD sequence ion intensity prediction model, the separation of the PCC values (0.994 *versus* 0.838) is even larger, presumably due to the higher accuracy of our prediction model (see Supplemental Fig. S4.2).

**Fig. 7.**
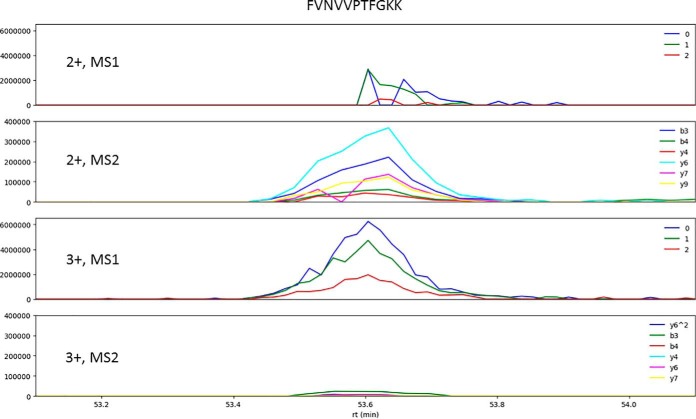
**Extracted ion chromatograms of FVNVVPTFGKK 2+ and 3+ precursor ions from a DIA experiment.** The first and the third panels are from survey or MS1 scans; they have the same vertical scale. The labels are 0, the precursor's monoisotopic peak; 1 and 2, the first and second isotopic peaks. The second and fourth panels are from MS2 scans with the corresponding selection windows; they also have the same vertical scale. The labels indicate the six most abundant (determined by the use of the HCD sequence ion intensity prediction model) product ions used for extraction.

To demonstrate the utility of charge state distribution prediction for DIA analysis, a peptide (FVNVVPTFGKK) was selected and its ion chromatograms were extracted from a DIA data file (Fig. 7). Our charge state distribution prediction model predicts that the 2+ precursor charge state (0.32) is X2 less abundant that 3+ (0.68).

Even the 3+ precursor ion has greater abundance, its MS2 product ion abundance levels are low (*bottom panel*, Fig. 7), and only three of the six product ions display signals and they are very weak. It is therefore more confident to use the MS1 XICs for charge state 3+ and MS2 XICs for charge state 2+ for the peptide identification and quantification.

A web service for LC-MS/MS property prediction is provided (see Supplemental Materials Section S5). The trained models, training and testing data, and associated Python codes can be downloaded from Zenodo (see Supplemental Materials Section S6).

## DISCUSSION

Deep learning models have been surveyed for prediction of three key LC-MS/MS properties of peptides: iRT, MS1 charge state distribution, and HCD sequence ion intensities. The three properties characterize peptide behaviors at the three distinct levels of experiment: chromatography, MS1 or precursor detection, and MS/MS or fragmentation of a precursor. The deep learning models share the same peptide sequence encoding strategy (additional charge encoding for spectrum prediction) and structure of the first two layers of recurrent neural network. The output layers and the corresponding activation functions were chosen to reflect the need for the different types of output dimensionality.

Those models provide superior performance. The iRT prediction is ∼28% more accurate compared with that of the DeepRT model(3). The 95% confidence interval for iRT prediction of Prosit (14) is nearly twice as small as that of this work. However, training and testing data of Prosit came from pools of synthetic peptides on the order of 1000 with equal abundance, whereas data used for this study came from a complex sample of cell lysate. It is indeed useful to investigate the impact of sample complexity, gradient length on the iRT prediction accuracy. For the charge state distribution prediction, a median test PCC of 0.997 was achieved. The performance of our HCD sequence ion intensity prediction model outperforms that of the pDeep model (12). Our model is also able to predict peptide sequence length of 40 compared with 20 for pDeep and allows for common peptide modifications. The superior performance of our models may come from two factors. First, our models are simpler with minimal feature engineering. More complicated feature engineering (as the case in the pDeep model) may set constraints on the model to fit data and to generalize well. Second, our training datasets may be more homogeneous.

Prosit's fragment ion intensity model uses bidirectional gated recurrent unit (14), which is less sophisticated and more efficient to train than the LSTM model used in this study. All the prediction models are simpler enough, and the computation effort is not a major practical issue. The other difference is that our spectrum prediction model (and that for pDeep) uses a time distributed layer in combination with a masking layer to discard the useless parameters and output dimensions. The Prosit model sets the useless output labels to −1.

The authors of Prosit (14) claimed to have achieved a median PCC of 0.99 between the experimental and predicted spectra. However, as pointed out by the authors of pDeep, the agreement among their experimental spectra was the PCC of 0.981, which was considered as the upper limit of any prediction model. Tiwary *et al.* (15) developed two models, DeepMass:Prism and wiNNer. DeepMass:Prism, an LSTM-based model, achieves a median PCC of 0.925 for HCD spectra.

Our models with current trained parameter values are practically useful in the right context: for example the iRT predicted values are only useful in combination of the right iRT to RT calibration. The current prediction model for HCD sequence ion intensities does not account for internal fragmentation and neutral loss from fragment ions. Extension to include simple neutral loss can be accomplished with the expansion of the label dimension (See Supplemental Fig. S2.2).

Our models for prediction of iRT and charge state distribution may have learned major portions of the corresponding data generation distributions because the labels (the measured iRTs and charge state distributions) contain limited information. More training data may not improve their performance. The model for HCD sequence ion intensity prediction on the other hand may be further improved given more data.

We also proposed two encoding schemes to featurize modifications: (A) if a modification occurs on many different types of amino acid residues, a separate feature is created or (B) if a modification occurs in only on one or two types of amino acid residues, the modified residues are used as features. As illustrated in Supplemental Figs. S2.1 and S2.2, featurization Scheme (A) will increase the space occupied by the separately coded modifications in the sequence length dimensions in both sequence code and label. The Scheme (B) entries will increase the feature dimension in sequence code. An optimal design may be a trade-off of the two conflicting effects to minimize the dimensions for both sequence code and label.

Broad applications of those models are subject to future studies. The models can supply DIA data extraction algorithms with predicted retention times, charge state distributions, and spectra for peptides not previously detected. Enhancement of search engines' theoretical fragment pattern generation, expansion of spectral libraries for spectral library search, selected ion monitoring (SRM), MRM, PRM method development can all benefit from the models' capabilities.

## Data Availability

Training and testing data, trained models, prediction results, sample codes for training and accessing data are provided in the following link: https://zenodo.org/record/2652602#.XP67VlxKi70.

## Supplementary Material

Supplemental_Materials_LCMSMS_Property_Prediction

## Footnotes

^1^ The abbreviations used are:

LC-MS/MS: liquid chromatography - tandem mass spectrometry
DIA: data independent acquisition
CNN: convolutional neural networks
ETD: electron transfer dissociation
GPU: graphic processing unit
HCD: higher-energy collisional dissociation
iRT: Indexed retention time
LSTM: long-short term memory
MRM: multiple reaction monitoring
MS/MS: tandem mass spectrometry
MS1: the first level of mass analysis
MS2: the second level of mass analysis
NIST: National Institute of Standards and Technology
PRM: parallel reaction monitoring
PCC: Pearson correlation coefficient
RT: retention time
SRM: selected ion monitoring
XIC: extracted ion chromatogram.
