# Supplementary material for: Prediction of LC-MS/MS Properties of Peptides from Sequence by Deep Learning
Source: Mol Cell Proteomics. 2019 Jun 27;18(10):2099–107. doi: 10.1074/mcp.TIR119.001412 (PMC6773555; doi:10.1074/mcp.TIR119.001412)
Supplement: Supplemental_Materials_LCMSMS_Property_Prediction [file 143901_2_supp_345904_pswq56.pdf]

Supplemental Materials for

Prediction of LCMSMS properties of peptides from sequence by deep learning

Shenheng Guan [1,2,*] , Michael F. Moran [2,3] , and Bin Ma [1]

[1] David R. Cheriton School of Computer Science, University of Waterloo, Waterloo, N2L 3G1, Canada
[2] Program in Cell Biology and SPARC BioCentre, Hospital for Sick Children, 686 Bay St, Toronto, ON, M5G 0A4, Canada
[3] Department of Molecular Genetics, University of Toronto, 686 Bay St, Toronto, ON, M5G 0A4, Canada
[*] Corresponding Author: shenheng.guan@uwaterloo.ca

S1. RT vs iRT calibration

In this section, we illustrate the process for calibration of index retention time (iRT) against experimental retention time.

In the iRT strategy(1), a set of synthetic peptides were chosen as retention time standards and each of the standard peptides was given an iRT value. The iRT standard peptides are spiked into the sample and their RT and iRT value pairs (or anchor or milestone points) can be used to establish the RT vs iRT calibration function. The iRT values of other peptides in the sample can be calculated using the calibration function. If a sample does not contain the iRT peptides, the CiRT (conserved internal RT) method(2) may be used. CiRT peptides are peptides consistently present in the samples and their retention times have been calibrated against iRT. RT vs iRT calibration curves are typically nonlinear, but monotonic.

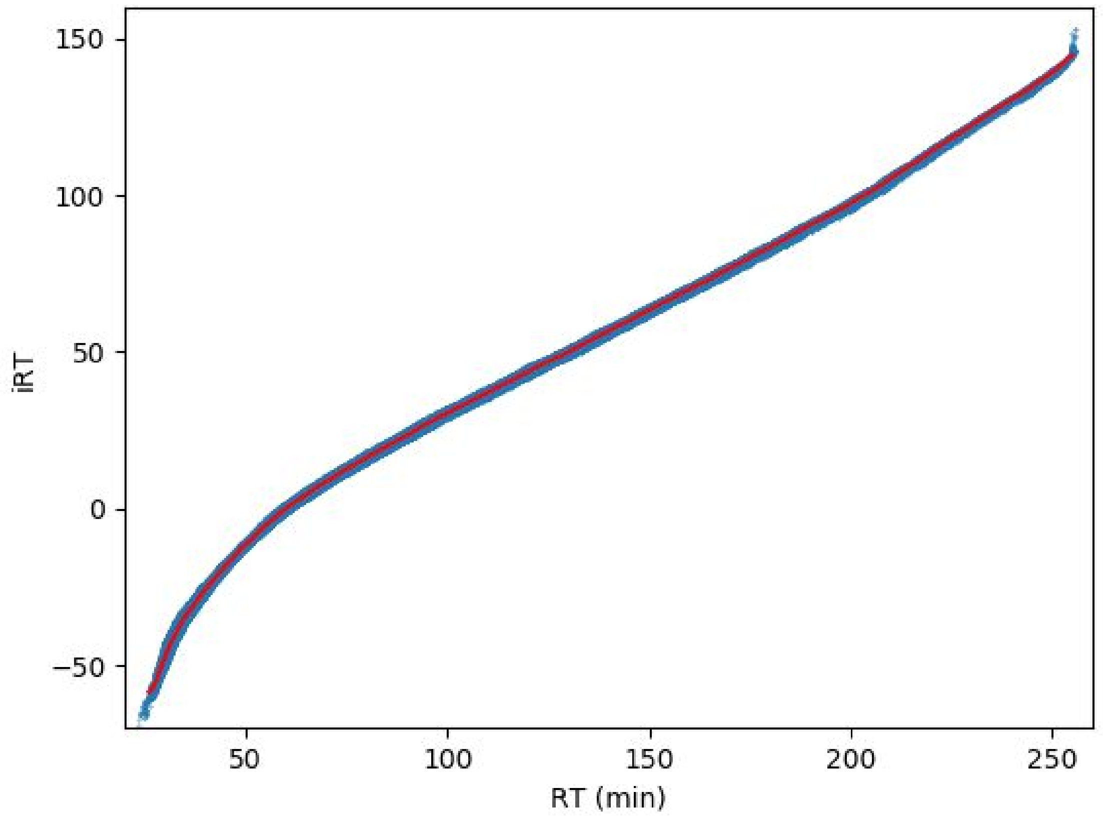

Supplemental Figure S1. The RT vs iRT polt for the data used in the iRT prediction model (blue dots). The red curve is produced by a K-mean regressor with k=500.

Calibration of iRT requires robust nonlinear regression, such as segmented linear fitting algorithm(3). In principle, one can use any suitable nonlinear regressors to summarize the RT vs iRT calibration curves. Here we demonstrate it with a K-mean regressor in Figure S1.

Due to the monotonicity of the RT vs iRT curves (the inverse functions exist), iRTs can be used as surrogates for peer-to-peer retention time alignment. Multiple alignments are also made easier with the use of the iRT strategy.

S2. One-Hot Encoding of Peptide Sequence, Featurization of Modifications, and Label Structure of HCD Sequence Ion Intensity Prediction Model

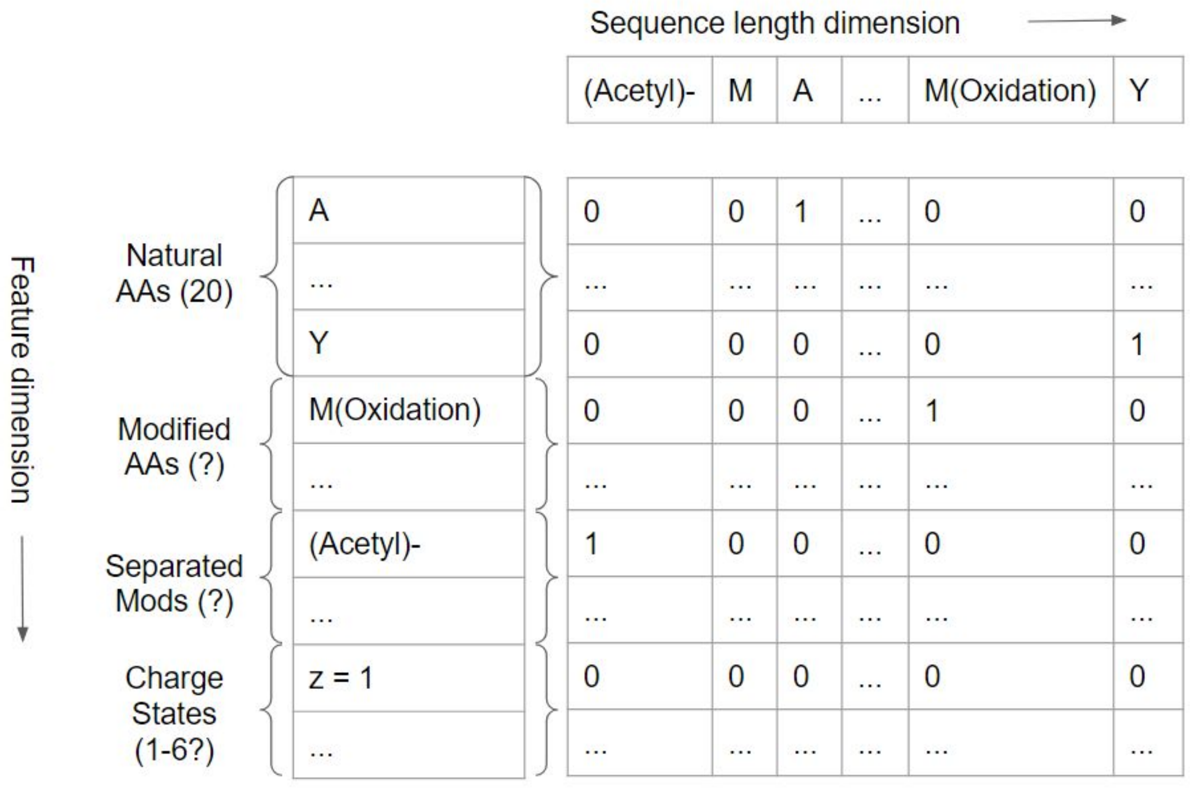

Supplemental Figure S2.1 Illustration of one-hot encoding of peptide sequence and featurization of modification. The peptide sequence of “(Acetyl)-MA...M(Oxidation)Y” is represented by a two-dimensional one-hot matrix with sequence and feature dimensions. In the current implementation, the maximal sequence length dimension is 40 and the feature dimension is 30 for HCD sequence ion intensity model, including 20 for natural amino acid residues, 2 for modified AAs ((Pyro-Glu)Q and M(Oxidation)), 2 for separated modifications ((Acetyl)- and (Carbamyl)), and 6 for charge states (1-6).

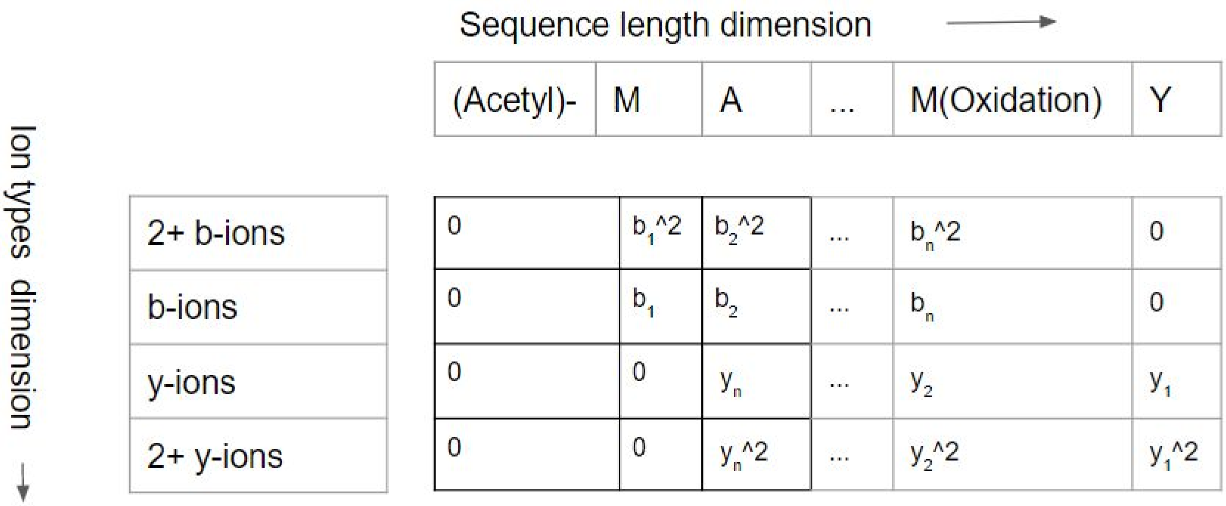

Supplemental Figure S2.2. Illustration of label structure of HCD sequence ion intensity prediction model. Unlike the pDeep model, both the b- and y- ions are aligned with peptide sequence in the model used in this work. Features for modification scheme A (discussion section) are set to zeros for training are skipped for prediction. The ends of b, b^2, y, and y^2 series are set to zero for training and discarded for prediction. For precursor charge states two or less, b^2 and y^2 series entries are also set to zero for training and discarded for prediction. The dimensionality of the label is 4 X (peptide sequence length (n + 1) + number of separated coded modifications in the sequence)). The useful output (such as spectral library entry) is 4 X n (by discarding zero-entries).

S3. Performance analysis of HCD sequence ion prediction

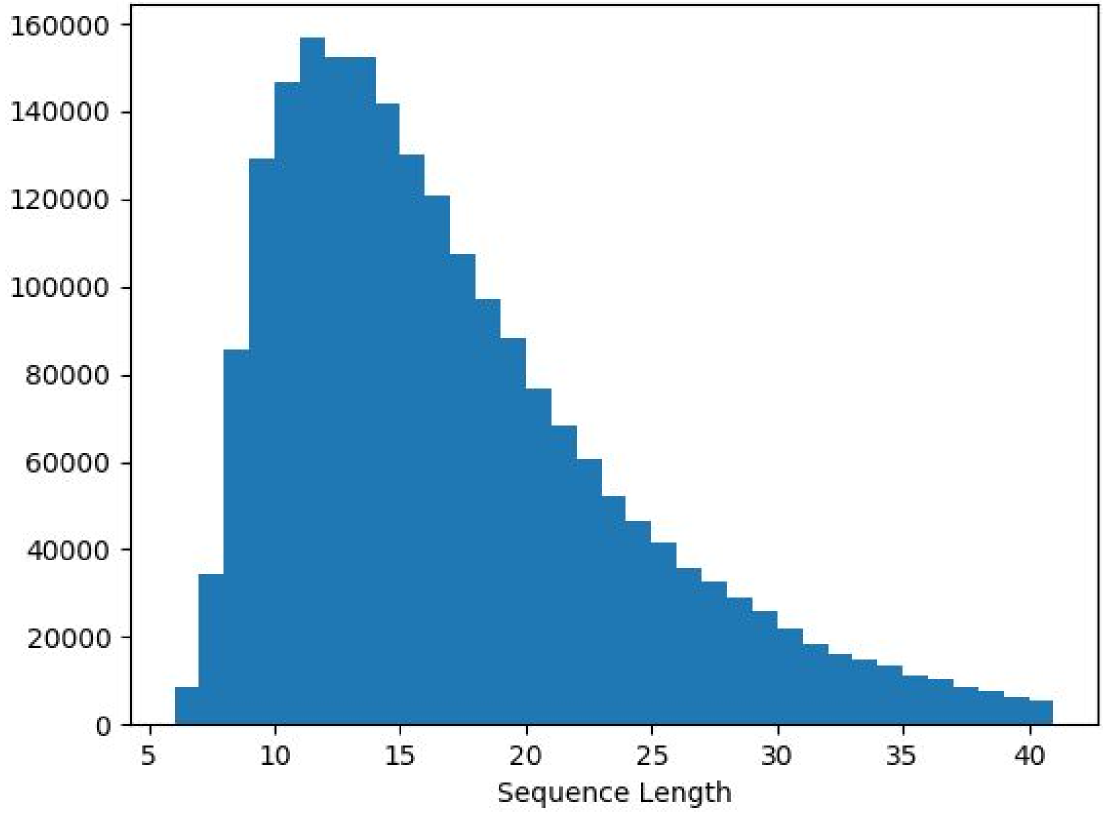

Figure S3. Length distribution of HCD sequence ion prediction dataset.

Median PCC/# of spectra	6 - 20 AAs	21 - 40 AAs
No modification	0.965/56,932	0.912/16,197
Has modification(s)	0.957/24,293	0.908/10,068

Table S3. Test performance breakdown of HCD sequence ion intensity prediction.

S4. Discrimination of isomeric peptides

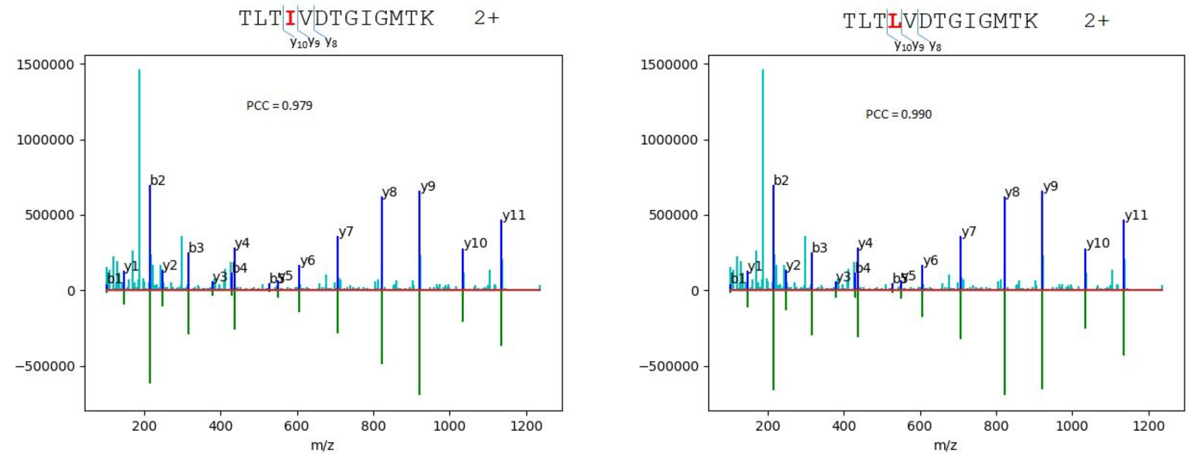

Supplemental Figure S4.1 Distinction of isoleucine/leucine isomeric peptides using HCD sequence ion intensity prediction (class (a) of isomeric peptides).

In Supplemental Figure S4.1, an experimental HCD spectrum was identified by the MSGF+ search engine as TLTIVDTGIGMTK 2+ ion of the putative heat shock protein HSP 90-alpha A4 (Q58FG1) or TLTLVDTGIGMTK 2+ ion of the Heat shock protein HSP 90-beta (P08238). Both sequences were assigned with the same EValue ($1.9X10^{-9}$, 1% FDR cutoff = 0.045). The PCC difference for matching between the experimental and predicted data were small (0.979 for TLTIVDTGIGMTK and 0.990 for TLTLVDTGIGMTK) if all sequence ions were used. However, if only some “local” ions were considered, such as y_8, y_9, and y_{10}, there is a clear difference in the relative intensities. The “local” PCC values for the three ions were 0.938 and 0.986 for TLTIVDTGIGMTK and TLTLVDTGIGMTK, respectively. The assignment of the leucine peptide of TLTLVDTGIGMTK was also supported by spectral count information. In the same raw data file, there are 197 spectra including the leucine peptide (9 spectra) were identified as the peptides of the protein (P08238) whereas only 22 spectra supporting the protein (Q58FG1). 9 out of the 22 spectra were assigned to the isoleucine peptide.

Example for isomeric peptide Class (b) is substitution of asparagine (N) with two glycines (GG). For the peptide pair of GGFFSFGDLTK and NFFSFGDLTK, it is almost impossible to distinguish them without using intensities of sequence ions. The experimental HCD spectrum was obtained from the ProteomeTools dataset (reference 20). The most commonly used sequence database search engines for peptide identification do not make a use of accurate fragment ion intensities and in most cases are incapable to discriminate one against another. With the pDeep model (reference 12), the pearson correlations between the experimental spectrum and the predicted ones allow for a clear choice of the correct GGFFSFGDLTK

sequence (PCC=0.98) over the incorrect NFFSFGDLTK (PCC=0.87), shown in Reference 11's Figure 6. (c) and (d).

Here, we perform the same analysis with our HCD sequence ion prediction model and the result is shown in Supplemental Figure S4.2.

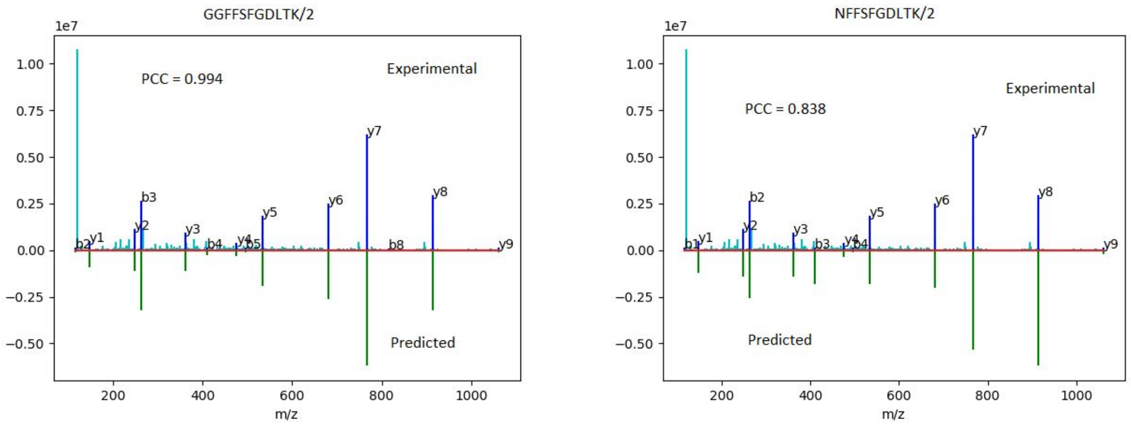

Supplemental Figure S4.2 Distinction of GG/N isomeric peptides using HCD sequence ion intensity prediction (class (b) of isomeric peptides). The wider spread in the pearson correlation (0.994 vs 0.838) may be due to the superior accuracy of our prediction model.

S5. Web service for LCMSMS property prediction

A rudimentary web service is provided at http://rsg-pc290.cs.uwaterloo.ca. Both username and password are “predict”. A user should prepare a tab-delimited file (with extension of “.txt”) containing two columns of data: peptide sequence in PSI format and the charge state. Please choose a unique file name without special characters including spaces. The column titles are “PSI_Sequence	Charge”.

Allowed modifications include: (Acetyl)-, (Carbamyl), (Gln->pyro-Glu)Q, C(Carbamidomethyl), M(Oxidation). (Acetyl)-, (Carbamyl), (Gln->pyro-Glu) are all peptide n-terminal modifications. All cysteines should be carbamidomethyled.

When the prediction service finishes the calculation, the user can download the result in a zip file containing two files: one with predicted iRT and charge state distribution and the other in the NIST spectral library format of msp. The predicted HCD sequence ion spectra can be displayed by spectral viewers such as NIST’s spectral search tool, illustrated by Figure S5.

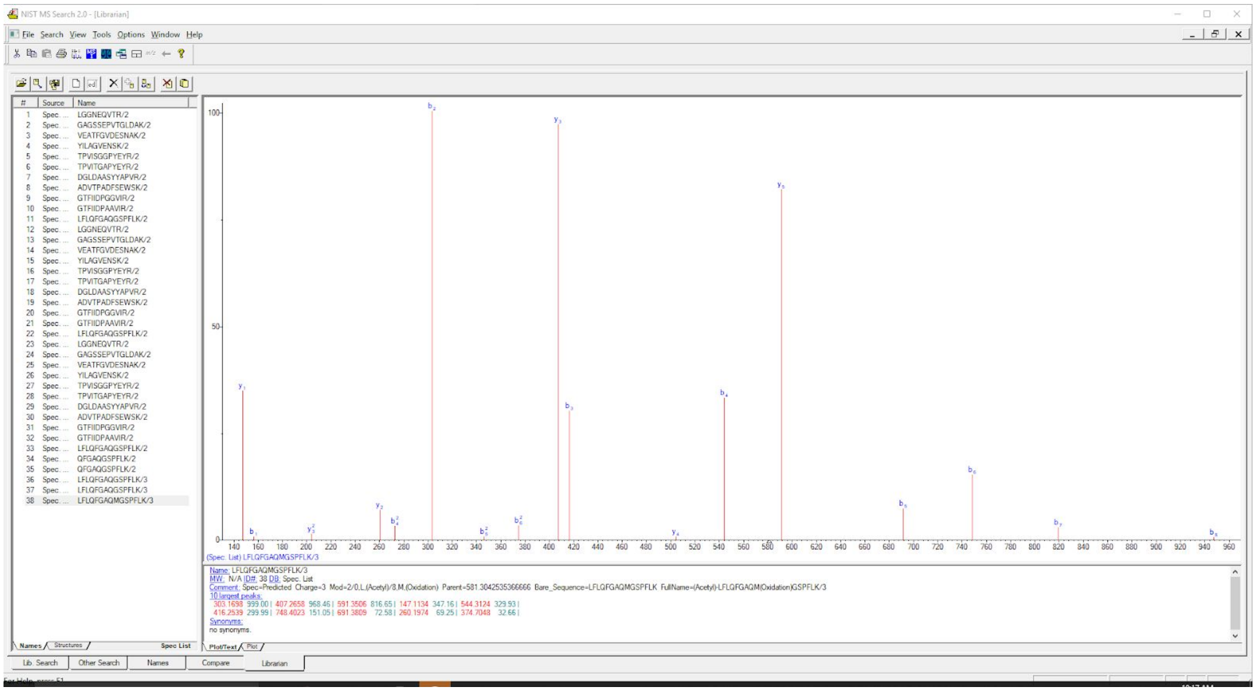

Figure S5. An HCD sequence ion spectrum displayed by NIST MS Search 2.0 software.

S6. Training data, models, prediction results, demonstration codes

The following information is included the Zenodo website as a zip file "LCMSMS_Pred_Supplemental_Material_section_S6.zip", 1GB of size.

1. A Python function for encoding peptide sequence (with PSI format modifications) one-hot-encode_peptide_ion.py
2. Training data for the three models (iRT/irt_reg_data.pickle, ChargeState/zfit_one_hot.pickle, and HCDspectrumPredict/ucsd_hcd_splib_2ndhalf.pickle)

3. Trained models in *.h5 format (iRT/irt_reg_data_filtered_bidirLSTM2_masking_model.h5, ChargeState/zfit_bidirLSTM2_masking_model.h5, HCDspectrumPredict/ucsd_hcd_splib_2ndhalf_model.h5)

4. Predicted data (iRT/irt_reg_data_filtered_bidirLSTM2_masking_result.pickle, ChargeState/zfit_bidirLSTM2_masking_result.pickle, and HCDspectrumPredict/ucsd_hcd_splib_2ndhalf_ions.pickle)

5. Codes to look at the results (iRT/plot_irt.py, ChargeState/plot_zfit_result.py, and HCDspectrumPredict/plot_predicted_spectrum.py

Supplemental Reference

1. Escher, C., Reiter, L., MacLean, B., Ossola, R., Herzog, F., Chilton, J., MacCoss, M. J., and Rinner, O. (2012) Using iRT, a normalized retention time for more targeted measurement of peptides. *Proteomics* 12, 1111–1121

2. Parker, S. J., Rost, H., Rosenberger, G., Collins, B. C., Malmström, L., Amodei, D., Venkatraman, V., Raedschelders, K., Van Eyk, J. E., and Aebersold, R. (2015) Identification of a Set of Conserved Eukaryotic Internal Retention Time Standards for Data-independent Acquisition Mass Spectrometry. *Mol. Cell. Proteomics* 14, 2800–2813

3. Bruderer, R., Bernhardt, O. M., Gandhi, T., and Reiter, L. (2016) High-precision iRT prediction in the targeted analysis of data-independent acquisition and its impact on identification and quantitation. *Proteomics* 16, 2246–2256
